# Crystal structures of bis­[2-(di­phenyl­phosphino­thio­yl)phen­yl] ether and bis­{2-[diphen­yl(selanyl­idene)phosphan­yl]phen­yl} ether

**DOI:** 10.1107/S1600536814023988

**Published:** 2014-11-19

**Authors:** Daron E. Janzen, Arianna M. Kooyman, Kayla A. Lange

**Affiliations:** a Department of Chemistry and Biochemistry, St Catherine University, St Paul, MN 55105, USA

**Keywords:** crystal structure, phosphine sulfide, phosphine selenide, functionalization of diphosphines, π–π inter­actions

## Abstract

The title compounds, C_36_H_28_OP_2_S_2_, (1), and C_36_H_28_OP_2_Se_2_, (2), exhibit remarkably similar structures although they are not isomorphous. The whole mol­ecule of compound (2) is generated by twofold symmetry, with the ether O atom located on the twofold axis. Both compounds have intra­molecular π–π inter­actions between terminal phenyl rings with centroid–centroid distances of 3.6214 (16) and 3.8027 (14) Å in (1) and (2), respectively. In the crystal of (1), short C—H⋯S hydrogen bonds link the mol­ecules, forming chains along [001], while in (2) there are no analogous C—H⋯Se inter­actions present.

## Abstract 2

The title compounds exhibit remarkably similar structures although they are not isomorphous. In the crystal of the sulfur analogue, mol­ecules are linked *via* C—H⋯S hydrogen bonds, forming chains along [001], while in the crystal of the selenium analogue, there are no C—H⋯Se hydrogen bonds present.

## Chemical context   

The ligand bis­[2-(di­phenyl­phosphan­yl)phen­yl] ether (POP) and its congeners, including the more rigid Xantphos [(9,9-dimethyl-9*H*-xanthene-4,5-di­yl)bis­(di­phenyl­phosphane)], comprise a series of chelating diphosphines with a range of flexibility to accommodate variable bonding geometries at transition metals. Experimental and theoretical studies of metal complexes with diphosphines have shown a strong correlation between diphosphine bite angle and selectivity in catalytic transformations (Dierkes & van Leeuwen, 1999 ▶; Gathy *et al.*, 2011 ▶). Simple functionalization of these diphos­phines to form diphosphine dioxides, di­sulfides, and diselen­ides has permitted further tuning of the bonding of these ligands to metals by changing the bite-angle range as well as the electronic properties of these ligands. The π-accepting phospho­rous donor atoms of the parent diphosphines are profoundly altered with the addition of π-donor chalcogen donor atoms (Dairiki *et al.*, 2009 ▶). Chalcogen-modified diphosphine ligands have been utilized in strategies to tune the catalytic behavior of systems including the Pd^II^-catalysed hydro­amination of dienes (Jahromi *et al.*, 2012 ▶) and Ru^II^ transfer hydrogenation of aldehydes and ketones (Deb *et al.*, 2010 ▶). Hemilability, implicated in the selectivity and reactivity of some catalytic reactions (Braunstein *et al.*, 2001 ▶), can also result from the chalcogen functionalization of phosphines as well (Deb *et al.*, 2010 ▶).

Our inter­est in the application of chalcogen-substituted diphosphines to alter the electronic features of photoluminescent Cu^I^ sensor materials (Smith *et al.*, 2010 ▶) led us to study the solid-state structural features of the dichalcogen diphosphines, including the di­sulfide and diselenide of the ligand POP. We wanted to investigate the inter- and intra­molecular features that dominate the solid-state structural behavior of these ligands. The mol­ecular geometry and packing of these chalcogen diphosphines may strongly influence the geometric features of their *d* ^10^ metal complexes, as *d* ^10^ metals typically have poor stereochemical preferences. In this study, the structures obtained for bis­[2-(di­phenyl­phosphino­thio­yl)phen­yl] ether, (1), and bis­{2-[diphen­yl(selanyl­idene)phosphan­yl]phen­yl} ether, (2), are compared.
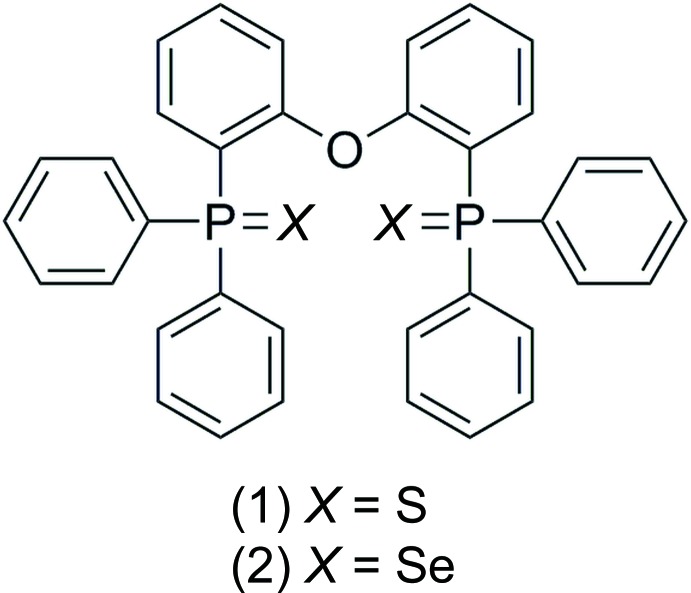


## Structural commentary   

**Figure 1:**
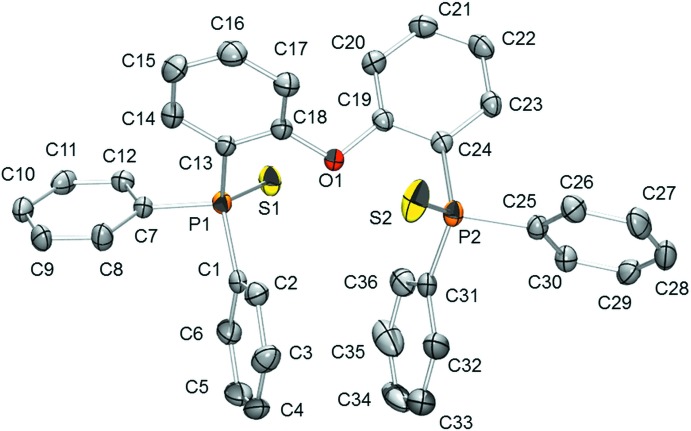
The mol­ecular structure of (1), showing the atom labelling and displacement ellipsoids drawn at the 50% probability level.

**Figure 3:**
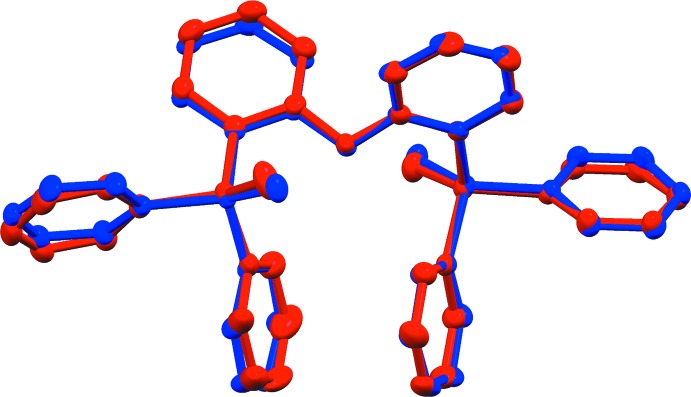
Structural overlay of (1) (red) and (2) (blue).

The mol­ecular structures of (1) and (2) are illustrated in Figs. 1 ▶ and 2 ▶, respectively. The P—S [1.9543 (8) and 1.9552 (9) Å] and P—Se [2.1125 (6) Å] bond lengths are consistent with covalent radii predictions as well as typical bond lengths for di­aryl­phosphine sulfides and selenides. Although these structures are not isomorphous, many intra­molecular features are remarkably alike despite the potentially flexible ether linkage of the diphosphine backbone. To demonstrate the similarity, several metrics were compared. The intra­molecular P⋯P distances [5.6452 (8) Å for (1); 5.669 (1) Å for (2)], the intra­molecular *E*⋯*E* distances [*E* = S 6.636 (1) Å for (1); *E* = Se 6.8246 (7) Å for (2)], and the *E*P⋯P*E* angles [158.29 (4)° for (1); 158.44 (2)° for (2)] all indicate a common geometry near the phospho­rous–chalcogen bonds. This similarity extends to the phenyl ring orientations. A structural overlap calculation of the pairwise atomic coordinates of all related atoms of (1) and (2) (except the chalcogens) reveals an r.m.s. deviation of only 0.214 Å over 39 atom pairs (Fig. 3 ▶).

**Figure 4:**
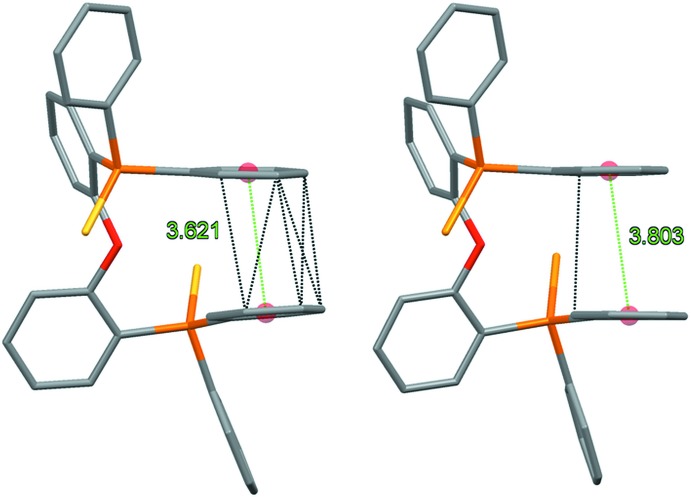
Intra­molecular π–π inter­actions in (1) and (2).

The largest differences in the intra­molecular features of (1) and (2) can be found in the closest approach of a pair of terminal phenyl rings, each bonded to different phospho­rous atoms (Fig. 4 ▶). In the structure of (2), the angle between mean planes formed by atoms C1–C6 and the twofold axis-related atoms C1–C6 of the same mol­ecule is 0.98 (12)°, with a centroid–centroid distance of 3.8027 (14) Å. The analogous relationship in the structure of (1), involving phenyl rings C1–C6 and C31–C36, is a dihedral angle of 6.52 (13)° and a centroid–centroid distance of 3.6214 (16) Å. The result of these differences is that in (2) there is only one C⋯C intra­molecular contact between these phenyl rings shorter than 3.6 Å, while in (1) there are six unique contacts that meet this criteria. Although these intra­molecular C⋯C contacts are slightly longer than the van der Waals radii sum of 3.4 Å, the additional C⋯C close-contacts in (1) may contribute to stronger intra­molecular π–π inter­actions between these phenyl rings compared to (2). The dihedral angles between the mean planes formed by the ether-linked phenyl groups [(C13–C18 and C19–C24) 76.83 (11)° for (1); (C13–C18 and the symmetry-related C13–C18 ring) 84.53 (11)° for (2)] also show a significant difference in the twist around the ether linkage.

## Supra­molecular features   

**Figure 5:**
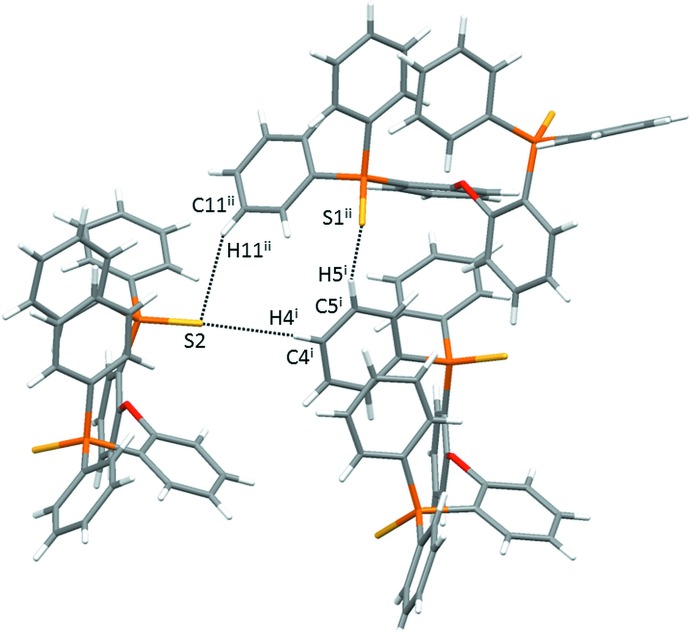
Inter­molecular C—H⋯S inter­actions in (1). [Symmetry codes: (i) *x* + 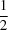, −*y* + 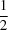, −*z* + 1; (ii) *x*, −*y* + 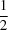, *z* + 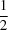.]

The inter­molecular features of (1) and (2) reveal additional differences between these seemingly similar structures. In the crystal of (1), most notably there are three unique inter­molecular C—H⋯S inter­actions (Table 1 ▶) shorter than the sum of the van der Waals radii. Each mol­ecule participates as a C—H donor with two different S2 acceptors as well as one S1 acceptor (Table 1 ▶ and Fig. 5 ▶). As such, each mol­ecule is involved in C—H⋯S inter­molecular inter­actions with three other unique mol­ecules. In the crystal of (2), no analogous C—H⋯Se inter­molecular inter­actions are present.

**Figure 6:**
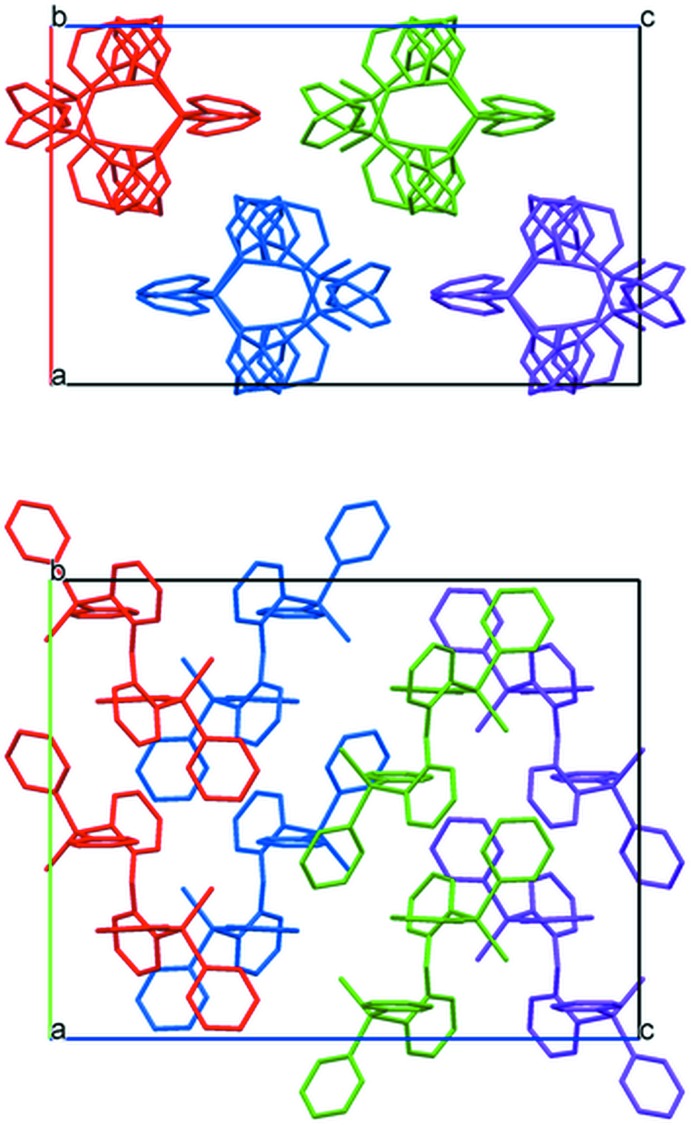
Crystal packing of (1), viewed along [010] (above) and [100] (below). Color to highlight mol­ecules packing within columns.

**Figure 7:**
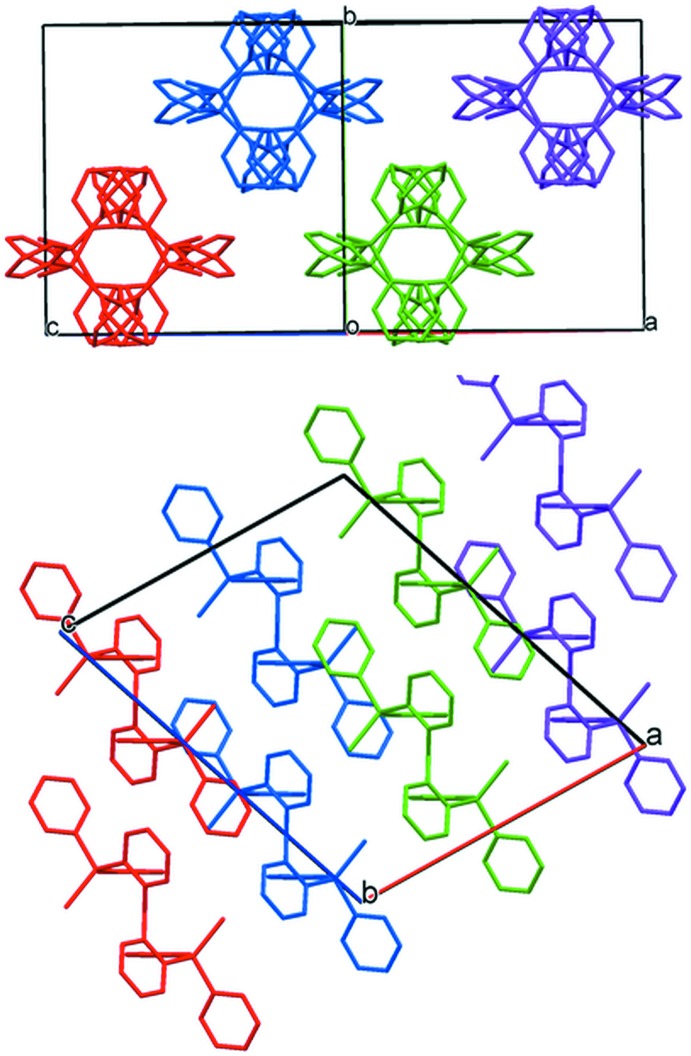
Crystal packing of (2) viewed along [101] (above) and [010] (below). Color to highlight mol­ecules packing within columns.

Both structures show that several inter­molecular C—H⋯π contacts less than *ca* 3.0 Å are present but these are likely to play a weak role in packing inter­actions [see Table 1 ▶ for (1) and Table 2 ▶ for (2)]. Mol­ecules of (1) stack in columns parallel to [010] (Fig. 6 ▶). The intra­molecular π–π stacking inter­actions of (1) are all aligned perpendicular to the column stacking axis. Mol­ecules of (2) stack in columns parallel to [101] (Fig. 7 ▶) with intra­molecular π–π stacking perpendicular to the column stacking vector.

## Database survey   

The Cambridge Structural Database (Version 5.35; Groom & Allen, 2014 ▶) contains several closely related phosphine sulfide structures, including Xantphos di­sulfide (Jahromi *et al.*, 2012 ▶), POP mono­sulfide (Deb *et al.*, 2010 ▶), and POP dioxide (Deb & Dutta, 2010 ▶). As the xanthene backbone of the diphosphine linkage is more sterically constrained compared with the ether linkage of POP, the Xantphos di­sulfide structure forces the intra­molecular S⋯S [4.207 (1) Å] and P⋯P [4.984 (1) Å] distances to be much shorter compared with (1). The structure of POP mono­sulfide is also very different from (1), as intra­molecular phenyl ring inter­actions are present but these involve a terminal phenyl ring and a bridging phenyl ring rather than two terminal phenyl rings as in (1). POP dioxide adopts a conformation unlike (1) or (2), as the P—O bond vectors are closer to anti­parallel [intra­molecular OP⋯P—O angles of 37.0 (6)°]. Considering metal complexes of related ligands, the structures of only two ruthenium(II) complexes (Deb *et al.*, 2010 ▶), three palladium(II) complexes (Milheiro & Faller, 2011 ▶; Saikia *et al.*, 2012 ▶), and one rhodium(I) complex (Faller *et al.*, 2008 ▶) have been reported with Xantphos sulfide or POP sulfide. The structure of only one palladium(II) complex of Xantphos di­sulfide (Jahromi *et al.*, 2012 ▶) is reported. POP or Xantphos selenide structures are even rarer, as only one copper(I) complex of POP selenide is reported (Venkateswaran *et al.*, 2007*b* ▶). No structures to date have been reported with diselenides of POP or Xantphos.

## Synthesis and crystallization   

Compounds (1) and (2) were prepared using a reported procedure (Venkateswaran *et al.*, 2007*a* ▶). Crystals of each sample were obtained by diffusion of diethyl ether into a concentrated di­chloro­methane solution.

## Refinement   

Crystal data, data collection and structure refinement details are summarized in Table 3 ▶. H atoms were placed in calculated positions and refined in the riding-model approximation: C—H = 0.95 Å with *U_iso_*(H) = 1.2*U_eq_*(C).

A small number of low-angle reflections [nine for (1) and five for (2)] were missing from these high-quality data sets due to the arrangement of the instrument with a conservatively sized beam stop and a fixed-position detector. The large number of reflections in the data sets (and the Fourier-transform relationship of intensities to atoms) ensures that no particular bias was thereby introduced into this routine structure determination.

## Supplementary Material

Crystal structure: contains datablock(s) 1, 2, general. DOI: 10.1107/S1600536814023988/su5010sup1.cif

Structure factors: contains datablock(s) 1. DOI: 10.1107/S1600536814023988/su50101sup2.hkl

Structure factors: contains datablock(s) 2. DOI: 10.1107/S1600536814023988/su50102sup3.hkl

Supporting information file. DOI: 10.1107/S1600536814023988/su50101sup4.cml

Supporting information file. DOI: 10.1107/S1600536814023988/su50102sup5.cml

CCDC references: 1031850, 1031849

Additional supporting information:  crystallographic information; 3D view; checkCIF report

## Figures and Tables

**Figure 2:**
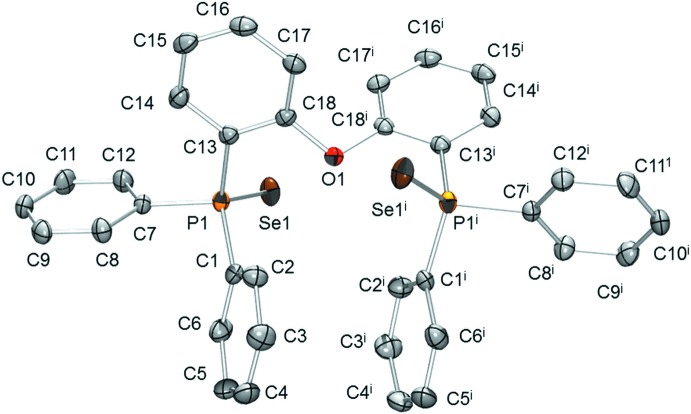
The mol­ecular structure of (2), showing the atom labelling and displacement ellipsoids drawn at the 50% probability level. [Symmetry code: (i) −*x*, *y*, −*z* + 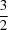.]

**Table 1:** Hydrogen-bond geometry (, ) for (1) *Cg*4 is the centroid of ring C19C24.

*D*H*A*	*D*H	H*A*	*D* *A*	*D*H*A*
C11H11S2^i^	0.95	2.82	3.696(2)	153
C4H4S2^ii^	0.95	2.94	3.698(3)	138
C5H5S1^iii^	0.95	2.93	3.796(3)	152
C9H9*Cg*4^iv^	0.95	2.94	3.598(3)	127

Symmetry codes: (i) 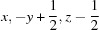; (ii) 
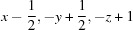
; (iii) 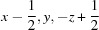; (iv) 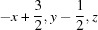.

**Table 2:** Hydrogen-bond geometry (, ) for (2) *Cg*2 and *Cg*3 are the centroids of rings C7C12 and C13C18, respectively.

*D*H*A*	*D*H	H*A*	*D* *A*	*D*H*A*
C5H5*Cg*2^i^	0.95	2.63	3.546(3)	161
C9H9*Cg*3^ii^	0.95	2.94	3.676(3)	135

Symmetry codes: (i) 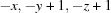; (ii) 
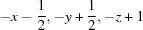
.

**Table 3:** Experimental details

	(1)	(2)
Crystal data		
Chemical formula	C_36_H_28_OP_2_S_2_	C_36_H_28_OP_2_Se_2_
*M*_r_	602.64	696.44
Crystal system, space group	Orthorhombic, *P* *b* *c* *a*	Monoclinic, *C*2/*c*
Temperature (K)	173	173
*a*, *b*, *c* ()	14.1161(9), 18.0874(12), 23.1986(16)	14.0964(15), 13.0854(13), 17.5918(18)
, , ()	90, 90, 90	90, 109.226(8), 90
*V* (^3^)	5923.1(7)	3064.0(6)
*Z*	8	4
Radiation type	Mo *K*	Mo *K*
(mm^1^)	0.32	2.55
Crystal size (mm)	0.52 0.24 0.12	0.80 0.12 0.12

Data collection		
Diffractometer	Rigaku XtaLAB mini	Rigaku XtaLAB mini
Absorption correction	Multi-scan (*REQAB*; Rigaku, 1998 ▶)	Multi-scan (*REQAB;* Rigaku, 1998 ▶)
*T*_min_, *T*_max_	0.718, 0.963	0.556, 0.737
No. of measured, independent and observed [*I* > 2(*I*)] reflections	54343, 6050, 4671	15840, 3521, 2958
*R*_int_	0.073	0.045
(sin /)_max_ (^1^)	0.625	0.649

Refinement		
*R*[*F* ^2^ > 2(*F* ^2^)], *wR*(*F* ^2^), *S*	0.045, 0.101, 1.07	0.032, 0.066, 1.07
No. of reflections	6050	3521
No. of parameters	370	186
H-atom treatment	H-atom parameters constrained	H-atom parameters constrained
_max_, _min_ (e ^3^)	0.37, 0.33	0.40, 0.41

Computer programs: *CrystalClear-SM Expert* (Rigaku Americas and Rigaku, 2011 ▶), *SIR2004* (Burla *et al.*, 2005 ▶), *SHELXL2013* (Sheldrick, 2008 ▶), *ORTEP-3 for Windows* (Farrugia, 2012 ▶) and *CrystalStructure* (Rigaku, 2010 ▶).

## supplementary crystallographic information

### Crystal data

**Table:**

C_36_H_28_OP_2_Se_2_	*F*(000) = 1400
*M**_r_* = 696.44	*D*_x_ = 1.510 Mg m^−^^3^
Monoclinic, *C*2/*c*	Mo *K*α radiation, λ = 0.71075 Å
*a* = 14.0964 (15) Å	Cell parameters from 13788 reflections
*b* = 13.0854 (13) Å	θ = 3.1–27.6°
*c* = 17.5918 (18) Å	µ = 2.55 mm^−^^1^
β = 109.226 (8)°	*T* = 173 K
*V* = 3064.0 (6) Å^3^	Prism, colorless
*Z* = 4	0.80 × 0.12 × 0.12 mm

### Data collection

**Table:**

Rigaku XtaLAB mini diffractometer	3521 independent reflections
Radiation source: normal-focus sealed tube	2958 reflections with *I* > 2σ(*I*)
Detector resolution: 6.849 pixels mm^-1^	*R*_int_ = 0.045
ω scans	θ_max_ = 27.5°, θ_min_ = 3.1°
Absorption correction: multi-scan (*REQAB;* Rigaku, 1998)	*h* = −18→18
*T*_min_ = 0.556, *T*_max_ = 0.737	*k* = −16→16
15840 measured reflections	*l* = −22→22

### Refinement

**Table:**

Refinement on *F*^2^	0 restraints
Least-squares matrix: full	Hydrogen site location: inferred from neighbouring sites
*R*[*F*^2^ > 2σ(*F*^2^)] = 0.032	H-atom parameters constrained
*wR*(*F*^2^) = 0.066	*w* = 1/[σ^2^(*F*_o_^2^) + (0.0183*P*)^2^ + 4.7666*P*] where *P* = (*F*_o_^2^ + 2*F*_c_^2^)/3
*S* = 1.07	(Δ/σ)_max_ = 0.001
3521 reflections	Δρ_max_ = 0.40 e Å^−^^3^
186 parameters	Δρ_min_ = −0.41 e Å^−^^3^

### Special details

**Table:**

Geometry. All e.s.d.'s (except the e.s.d. in the dihedral angle between two l.s. planes) are estimated using the full covariance matrix. The cell e.s.d.'s are taken into account individually in the estimation of e.s.d.'s in distances, angles and torsion angles; correlations between e.s.d.'s in cell parameters are only used when they are defined by crystal symmetry. An approximate (isotropic) treatment of cell e.s.d.'s is used for estimating e.s.d.'s involving l.s. planes.

### Fractional atomic coordinates and isotropic or equivalent isotropic displacement parameters (Å^2^)

**Table:**

	*x*	*y*	*z*	*U*_iso_*/*U*_eq_	
Se1	0.15497 (2)	0.31054 (2)	0.63637 (2)	0.02846 (8)	
P1	−0.00071 (4)	0.28040 (4)	0.58870 (3)	0.01801 (12)	
O1	0.0000	0.35271 (16)	0.7500	0.0191 (4)	
C1	−0.03973 (16)	0.15982 (16)	0.61952 (12)	0.0201 (5)	
C2	−0.10828 (17)	0.15221 (18)	0.66065 (14)	0.0267 (5)	
H2	−0.1341	0.2123	0.6770	0.032*	
C3	−0.13892 (19)	0.0569 (2)	0.67788 (15)	0.0336 (6)	
H3	−0.1859	0.0521	0.7060	0.040*	
C4	−0.10223 (19)	−0.03063 (19)	0.65480 (15)	0.0338 (6)	
H4	−0.1242	−0.0956	0.6665	0.041*	
C5	−0.0331 (2)	−0.02409 (19)	0.61439 (15)	0.0333 (6)	
H5	−0.0075	−0.0846	0.5985	0.040*	
C6	−0.00137 (18)	0.07029 (18)	0.59724 (14)	0.0284 (5)	
H6	0.0468	0.0745	0.5702	0.034*	
C7	−0.04357 (15)	0.27085 (16)	0.47944 (12)	0.0189 (4)	
C8	−0.13290 (16)	0.22060 (18)	0.43821 (13)	0.0238 (5)	
H8	−0.1715	0.1900	0.4673	0.029*	
C9	−0.16556 (16)	0.21505 (18)	0.35504 (14)	0.0264 (5)	
H9	−0.2261	0.1801	0.3272	0.032*	
C10	−0.11013 (18)	0.26041 (18)	0.31268 (14)	0.0276 (5)	
H10	−0.1332	0.2576	0.2555	0.033*	
C11	−0.02133 (19)	0.30990 (19)	0.35299 (14)	0.0312 (6)	
H11	0.0168	0.3407	0.3235	0.037*	
C12	0.01247 (17)	0.31487 (18)	0.43633 (13)	0.0255 (5)	
H12	0.0740	0.3484	0.4639	0.031*	
C13	−0.07964 (15)	0.37932 (16)	0.60936 (12)	0.0191 (4)	
C14	−0.15170 (16)	0.43227 (17)	0.54724 (14)	0.0237 (5)	
H14	−0.1613	0.4148	0.4928	0.028*	
C15	−0.20897 (16)	0.50950 (17)	0.56408 (15)	0.0269 (5)	
H15	−0.2571	0.5447	0.5213	0.032*	
C16	−0.19615 (16)	0.53555 (17)	0.64311 (15)	0.0267 (5)	
H16	−0.2361	0.5880	0.6545	0.032*	
C17	−0.12510 (16)	0.48518 (17)	0.70579 (14)	0.0227 (5)	
H17	−0.1160	0.5031	0.7601	0.027*	
C18	−0.06746 (15)	0.40848 (16)	0.68848 (13)	0.0188 (4)	

### Atomic displacement parameters (Å^2^)

**Table:**

	*U*^11^	*U*^22^	*U*^33^	*U*^12^	*U*^13^	*U*^23^
Se1	0.01582 (11)	0.04058 (16)	0.02624 (13)	−0.00167 (10)	0.00320 (9)	−0.00400 (11)
P1	0.0159 (3)	0.0209 (3)	0.0167 (3)	−0.0006 (2)	0.0045 (2)	0.0003 (2)
O1	0.0205 (10)	0.0157 (10)	0.0196 (10)	0.000	0.0047 (9)	0.000
C1	0.0214 (11)	0.0192 (11)	0.0178 (10)	0.0017 (8)	0.0037 (9)	0.0022 (8)
C2	0.0281 (12)	0.0227 (12)	0.0325 (13)	−0.0018 (9)	0.0142 (11)	−0.0006 (10)
C3	0.0355 (14)	0.0317 (15)	0.0396 (15)	−0.0068 (11)	0.0202 (12)	0.0012 (11)
C4	0.0409 (14)	0.0209 (13)	0.0363 (14)	−0.0047 (11)	0.0084 (12)	0.0064 (10)
C5	0.0453 (15)	0.0202 (13)	0.0329 (13)	0.0092 (11)	0.0108 (12)	0.0022 (10)
C6	0.0312 (13)	0.0279 (13)	0.0281 (12)	0.0063 (10)	0.0126 (10)	0.0038 (10)
C7	0.0194 (10)	0.0193 (11)	0.0172 (10)	−0.0004 (8)	0.0047 (8)	0.0005 (8)
C8	0.0191 (11)	0.0285 (13)	0.0233 (11)	−0.0044 (9)	0.0064 (9)	−0.0018 (9)
C9	0.0192 (11)	0.0282 (13)	0.0272 (12)	−0.0004 (9)	0.0013 (9)	−0.0034 (10)
C10	0.0347 (13)	0.0273 (13)	0.0178 (11)	0.0008 (10)	0.0047 (10)	0.0019 (9)
C11	0.0386 (14)	0.0326 (14)	0.0244 (12)	−0.0083 (11)	0.0129 (11)	0.0066 (10)
C12	0.0253 (11)	0.0257 (12)	0.0246 (11)	−0.0089 (10)	0.0071 (9)	0.0002 (10)
C13	0.0170 (10)	0.0166 (11)	0.0225 (11)	−0.0018 (8)	0.0049 (9)	0.0009 (9)
C14	0.0208 (11)	0.0234 (12)	0.0240 (11)	−0.0027 (9)	0.0035 (9)	0.0032 (9)
C15	0.0191 (11)	0.0208 (12)	0.0353 (13)	0.0007 (9)	0.0018 (10)	0.0059 (10)
C16	0.0199 (11)	0.0184 (12)	0.0411 (14)	0.0014 (9)	0.0091 (10)	−0.0010 (10)
C17	0.0215 (11)	0.0192 (11)	0.0282 (12)	−0.0038 (9)	0.0090 (10)	−0.0029 (9)
C18	0.0167 (10)	0.0161 (11)	0.0232 (11)	−0.0028 (8)	0.0058 (9)	0.0027 (8)

### Geometric parameters (Å, º)

**Table:**

Se1—P1	2.1125 (6)	C8—C9	1.384 (3)
P1—C1	1.812 (2)	C8—H8	0.9500
P1—C7	1.819 (2)	C9—C10	1.379 (3)
P1—C13	1.820 (2)	C9—H9	0.9500
O1—C18	1.389 (2)	C10—C11	1.380 (3)
O1—C18^i^	1.389 (2)	C10—H10	0.9500
C1—C2	1.388 (3)	C11—C12	1.386 (3)
C1—C6	1.399 (3)	C11—H11	0.9500
C2—C3	1.385 (3)	C12—H12	0.9500
C2—H2	0.9500	C13—C18	1.398 (3)
C3—C4	1.372 (4)	C13—C14	1.405 (3)
C3—H3	0.9500	C14—C15	1.385 (3)
C4—C5	1.385 (4)	C14—H14	0.9500
C4—H4	0.9500	C15—C16	1.384 (3)
C5—C6	1.380 (3)	C15—H15	0.9500
C5—H5	0.9500	C16—C17	1.388 (3)
C6—H6	0.9500	C16—H16	0.9500
C7—C12	1.387 (3)	C17—C18	1.387 (3)
C7—C8	1.394 (3)	C17—H17	0.9500
			
C1—P1—C7	103.20 (10)	C10—C9—C8	119.9 (2)
C1—P1—C13	107.09 (10)	C10—C9—H9	120.1
C7—P1—C13	104.34 (10)	C8—C9—H9	120.1
C1—P1—Se1	114.95 (7)	C9—C10—C11	120.2 (2)
C7—P1—Se1	111.86 (7)	C9—C10—H10	119.9
C13—P1—Se1	114.30 (7)	C11—C10—H10	119.9
C18—O1—C18^i^	116.6 (2)	C10—C11—C12	120.2 (2)
C2—C1—C6	119.0 (2)	C10—C11—H11	119.9
C2—C1—P1	123.39 (17)	C12—C11—H11	119.9
C6—C1—P1	117.51 (17)	C11—C12—C7	120.0 (2)
C3—C2—C1	119.9 (2)	C11—C12—H12	120.0
C3—C2—H2	120.0	C7—C12—H12	120.0
C1—C2—H2	120.0	C18—C13—C14	117.4 (2)
C4—C3—C2	120.8 (2)	C18—C13—P1	120.67 (16)
C4—C3—H3	119.6	C14—C13—P1	121.89 (17)
C2—C3—H3	119.6	C15—C14—C13	121.1 (2)
C3—C4—C5	119.8 (2)	C15—C14—H14	119.5
C3—C4—H4	120.1	C13—C14—H14	119.5
C5—C4—H4	120.1	C16—C15—C14	120.1 (2)
C6—C5—C4	120.1 (2)	C16—C15—H15	119.9
C6—C5—H5	120.0	C14—C15—H15	119.9
C4—C5—H5	120.0	C15—C16—C17	120.2 (2)
C5—C6—C1	120.3 (2)	C15—C16—H16	119.9
C5—C6—H6	119.8	C17—C16—H16	119.9
C1—C6—H6	119.8	C18—C17—C16	119.4 (2)
C12—C7—C8	119.4 (2)	C18—C17—H17	120.3
C12—C7—P1	119.88 (16)	C16—C17—H17	120.3
C8—C7—P1	120.72 (16)	C17—C18—O1	120.62 (19)
C9—C8—C7	120.3 (2)	C17—C18—C13	121.83 (19)
C9—C8—H8	119.9	O1—C18—C13	117.40 (18)
C7—C8—H8	119.9		
			
C7—P1—C1—C2	−117.40 (19)	C9—C10—C11—C12	−0.4 (4)
C13—P1—C1—C2	−7.6 (2)	C10—C11—C12—C7	−0.6 (4)
Se1—P1—C1—C2	120.53 (18)	C8—C7—C12—C11	1.0 (4)
C7—P1—C1—C6	59.79 (19)	P1—C7—C12—C11	−178.38 (19)
C13—P1—C1—C6	169.57 (17)	C1—P1—C13—C18	71.55 (19)
Se1—P1—C1—C6	−62.27 (18)	C7—P1—C13—C18	−179.48 (17)
C6—C1—C2—C3	−1.0 (3)	Se1—P1—C13—C18	−57.00 (18)
P1—C1—C2—C3	176.11 (18)	C1—P1—C13—C14	−111.23 (18)
C1—C2—C3—C4	0.1 (4)	C7—P1—C13—C14	−2.2 (2)
C2—C3—C4—C5	0.5 (4)	Se1—P1—C13—C14	120.23 (16)
C3—C4—C5—C6	−0.1 (4)	C18—C13—C14—C15	−0.7 (3)
C4—C5—C6—C1	−0.9 (4)	P1—C13—C14—C15	−177.99 (17)
C2—C1—C6—C5	1.5 (3)	C13—C14—C15—C16	−0.3 (3)
P1—C1—C6—C5	−175.86 (18)	C14—C15—C16—C17	0.7 (3)
C1—P1—C7—C12	−145.39 (19)	C15—C16—C17—C18	−0.2 (3)
C13—P1—C7—C12	102.79 (19)	C16—C17—C18—O1	−176.18 (19)
Se1—P1—C7—C12	−21.3 (2)	C16—C17—C18—C13	−0.8 (3)
C1—P1—C7—C8	35.3 (2)	C18^i^—O1—C18—C17	−51.46 (16)
C13—P1—C7—C8	−76.5 (2)	C18^i^—O1—C18—C13	132.9 (2)
Se1—P1—C7—C8	159.41 (16)	C14—C13—C18—C17	1.2 (3)
C12—C7—C8—C9	−0.3 (3)	P1—C13—C18—C17	178.56 (16)
P1—C7—C8—C9	179.05 (17)	C14—C13—C18—O1	176.74 (17)
C7—C8—C9—C10	−0.7 (3)	P1—C13—C18—O1	−5.9 (3)
C8—C9—C10—C11	1.1 (4)		

Symmetry code: (i) −*x*, *y*, −*z*+3/2.

### Hydrogen-bond geometry (Å, º)

Cg2 and Cg3 are the centroids of rings C7–C12 and C13–C18, respectively.

**Table:**

*D*—H···*A*	*D*—H	H···*A*	*D*···*A*	*D*—H···*A*
C5—H5···*Cg*2^ii^	0.95	2.63	3.546 (3)	161
C9—H9···*Cg*3^iii^	0.95	2.94	3.676 (3)	135

Symmetry codes: (ii) −*x*, −*y*+1, −*z*+1; (iii) −*x*−1/2, −*y*+1/2, −*z*+1.
